# Real-time reverse transcription recombinase polymerase amplification (RT-RPA) assay for detection of cassava brown streak viruses

**DOI:** 10.1038/s41598-024-62249-y

**Published:** 2024-05-30

**Authors:** Florence M. Munguti, Dora C. Kilalo, Hillary K. Yegon, Isaac Macharia, Susan E. Seal, Agnes W. Mwango’mbe, Evans N. Nyaboga, Gonçalo Silva

**Affiliations:** 1 Department of Plant Science and Crop Protection, University Nairobi, Nairobi, Kenya; 2 Kenya Plant Health Inspectorate Service, Nairobi, Kenya; 3 Natural Resources Institute, Central Avenue, University of Greenwich, Chatham Maritime, ME4 4TB UK; 4 Department of Biochemistry, University of Nairobi, Nairobi, Kenya

**Keywords:** Cassava brown streak viruses, Early virus detection, Isothermal amplification, Rapid diagnosis, Reverse transcriptase recombinase polymerase amplification (RT-RPA), Microbiology, Molecular biology, Plant sciences

## Abstract

Cassava brown streak disease (CBSD) caused by *Cassava brown streak virus* (CBSV) and *Ugandan cassava brown streak virus* (UCBSV) is the most economically important viral disease of cassava. As cassava is a vegetatively propagated crop, the development of rapid and sensitive diagnostics would aid in the identification of virus-free planting material and development of effective management strategies. In this study, a rapid, specific and sensitive real-time reverse transcription recombinase polymerase amplification (RT-RPA) assay was developed for real-time detection of CBSV and UCBSV. The RT-RPA was able to detect as little as 2 pg/µl of purified RNA obtained from infected cassava leaves, a sensitivity equivalent to that obtained by quantitative real-time reverse transcription PCR (qRT-PCR), within 20 min at 37 °C. Further, the RT-RPA detected each target virus directly from crude leaf and stem extracts, avoiding the tedious and costly isolation of high-quality RNA. The developed RT-RPA assay provides a valuable diagnostic tool that can be adopted by cassava seed certification and virus resistance breeding programs to ensure distribution of virus-free cassava planting materials to farmers. This is the first report on the development and validation of crude sap-based RT-RPA assay for the detection of cassava brown streak viruses (UCBSV and CBSV) infection in cassava plants.

## Introduction

Cassava (*Manihot esculenta* Crantz) is a key food security crop and a major source of dietary calories for approximately 500 million people in sub-Saharan Africa^1–3^. Cassava productivity has, however been subjected to several biotic and abiotic challenges of which viral diseases are the most economically important. One of the major viral diseases of cassava is cassava brown streak disease (CBSD) that causes up to 100% yield losses in susceptible genotypes^4^. Cassava brown streak disease (CBSD) caused by two virus species namely *Cassava brown streak virus* (CBSV) and *Ugandan cassava brown streak virus* (UCBSV), genus *Ipomovirus*, family *Potyviridae* is the most economically important viral disease of cassava^5–12^. The disease is a major threat to cassava production in Africa contributing to food insecurity. Cassava brown streak disease (CBSD) is a threat to healthy propagation of cassava planting material especially in East and central Africa where the disease has been rampant. Annual economic losses of up to US$100 M have been reported due to CBSD infections^11,13^ as the brown corky necrosis in the storage roots render them unfit for consumption and unmarketable^14^. CBSD has negatively affected cassava production, challenging food security in many developing countries where cassava is largely cultivated.

The *Bemisia tabaci* species of whitefly that colonize cassava transmit the viruses that cause the devastating cassava brown streak disease in a semi persistent manner^12,15^. However, the vegetative propagation nature of cassava through cuttings majorly facilitates the spread and accumulation of viruses^16^. Effective management of viral diseases relies on accurate, rapid and sensitive detection of the causal agents of the disease. Virus indexing of cassava planting material for cassava brown streak viruses using highly sensitive diagnostic techniques is vital to support quarantine facilities and certification laboratories^17–20^. There is, therefore, a need to develop and improve the detection methods for timely decision on the health status of cassava planting material during certification and seed distribution programs^16,21^. This could greatly contribute to distribution of virus-free planting material and thus reducing the impact of the disease in cassava fields.

Early detection of cassava brown streak viruses is crucial when aiming to prevent the spread of CBSD through the use of infected stem cuttings as planting material. Visual inspection of CBSD symptoms has been considered unreliable since the symptoms vary according to cultivar and viral isolates^4,22^. Further, mite damage, nutrient disorders, age of the crop and other environmental factors have also been reported to contribute to inconsistent symptoms expression^4,23^. In addition, for some cassava cultivars, plant growth and development remain unaffected by the disease until during the harvest time when the storage roots are observed to have brown corky necrotic lesions. Furthermore, damage/necrotic lesions often remain unknown until harvest (at 9–12 months after planting), but has a significant impact on the usable yields and marketability of the storage roots^4,9^. This complicates the detection of cassava brown streak viruses based on visual inspection of symptoms on plant stems and leaves and often lead to further spread of the disease through movement of infected cassava cuttings^2,20^. Therefore, early detection of CBSD enhances better crop management and interventions.

Nucleic acid-based methods such as conventional reverse transcriptase polymerase chain reaction (RT-PCR)^17,24–26^ and quantitative real time reverse transcription PCR (qRT-PCR)^18,27^ have been developed and greatly accelerated specific detection of CBSV and UCBSV. Polymerase chain reaction (PCR)-based techniques allow detection of the viruses in asymptomatic cassava plants and have been reported to be more reliable and with greater sensitivity and specificity compared to serological techniques^28^. The primers developed by Mbanzibwa et al.^25^ have been widely used for simultaneous detection of CBSV and UCBSV in a two-step RT-PCR, followed by separation of the amplified products based on amplicon length in agarose gel electrophoresis. Use of multiplex RT-PCR assays reduces time and labour^29^. Quantitative real time reverse transcription PCR (qRT-PCR) has been used for absolute quantification of CBSV and UCBSV as a tool to accelerate breeding for CBSD resistance as well as a tool for high throughput virus detection^17,19,29^. The qRT-PCR provided greater sensitivity than both ELISA and conventional RT-PCR based assays. However, these PCR based methods have technical limitations. The methods depend on reliable power supply, sophisticated thermocycling equipment, trained personnel and are time consuming. In addition, these methods require careful preparation of the target nucleic acid from the sample and cannot be used for detection of viruses from crude extract of the samples due to known PCR inhibitors in crude samples^30–32^.

With the emergence of new molecular biology technologies, several isothermal amplification-based assays have recently been developed and widely adopted in many laboratories for rapid detection of plant pathogens. Amongst the different isothermal amplification techniques available^33^ loop-mediated isothermal amplification (LAMP) and recombinase polymerase amplification (RPA) have been extensively used for pathogen detection. Tomlinson et al*.*^34^ developed RT-LAMP test for detection of UCBSV and CBSV which demonstrated greater specificity and sensitivity than enzyme-linked immunosorbent assay (ELISA) and RT-PCR-based methods. LAMP amplifies specific regions of DNA under isothermal conditions between 60 and 65 °C and reactions can be carried out in simple system (e.g. water bath) or monitored in real time using either real-time PCR systems or portable fluorescence reading devices. This makes the technique easier to adapt in diagnostic laboratories with little resources. However, LAMP has some limitations that hinder its applicability such as the complex primer design and limited multiplexing potential^35^.

Recombinase polymerase amplification (RPA) has been used for detection of many plant viruses including Little cherry virus 2, Plum pox virus, Yam mosaic virus, amongst others^36–40^. The components of the RPA reaction are commercially available in a freeze-dried format and this has been shown to make the procedure easy to perform and improve the reaction stability^41^. RPA shares the same advantages as LAMP including quick reaction times and high specificity and sensitivity. However, RPA has a simpler primer (and probe) design and better multiplexing ability. RPA requires constant low temperature (37–42 °C) unlike the high temperature (60–65 °C) required in LAMP assays. The low reaction temperature of RPA compared to LAMP facilitates the implementation of RPA as a field-based method as amplification step can be performed without any special equipment. This has recently been demonstrated by Yilmaz and Batuman^42^ where they developed incubation equipment-free, body-heat-mediated reverse-transcription recombinase polymerase amplification combined with lateral flow assay (RT-RPA-LFA) technique to Tomato chlorotic spot virus (TCSV).

This study describes for the first time the development of a rapid and sensitive real-time reverse transcription recombinase polymerase amplification (RT-RPA) assay for specific detection of UCBSV and CBSV. The assay can be used to detect both viruses either directly from crude extracts obtained from stem and leaf samples or purified RNAs. The assay performance was compared with that of RT-PCR, qRT-PCR and RT-LAMP. The developed RT-RPA assay could improve early disease diagnosis and monitoring efforts and can be adopted by certification programs to ensure distribution of virus-free cassava planting materials to farmers.

## Results

### Initial evaluation of RPA primers for detection of CBSV and UCBSV

The presence of CBSV and UCBSV in the six-cassava reference and healthy control plant samples were confirmed by RT-PCR based on primers targeting the coat protein gene^9^. Six samples tested positive for UCBSV and CBSV as either single or as dual infections. Three samples (A, B and C) tested positive only for CBSV, whilst samples E and F were positive only for UCBSV (Table 1). Sample D showed dual infection and healthy control sample G tested negative for both viruses.

**Table 1:** List of reference samples used for the development of RPA assay.

Sample ID	Sample code	Status
197	A	Positive for CBSV only
FL2	B	Positive for CBSV only
CMK2	C	Positive for CBSV only
CMK36	D	Positive for both CBSV and UCBSV
180	E	Positive for both UCBSV only
206	F	Positive for both UCBSV only
AA10	G	Healthy Control

The RPA primers (Supplementary Table S1) targeting CP and CI regions of CBSV and UCBSV genomes, respectively, were initially evaluated using total RNAs extracted from the reference samples that had been confirmed positive for the two viruses using conventional RT-PCR. The results obtained using the designed RPA primers targeting CP and CI region were observed in agarose gels and selection was made based on the primer pairs that gave specific amplification for CBSV and UCBSV observed as clear bands on agarose gel (Supplementary Figs. S1 and S2).

**Figure 1:**
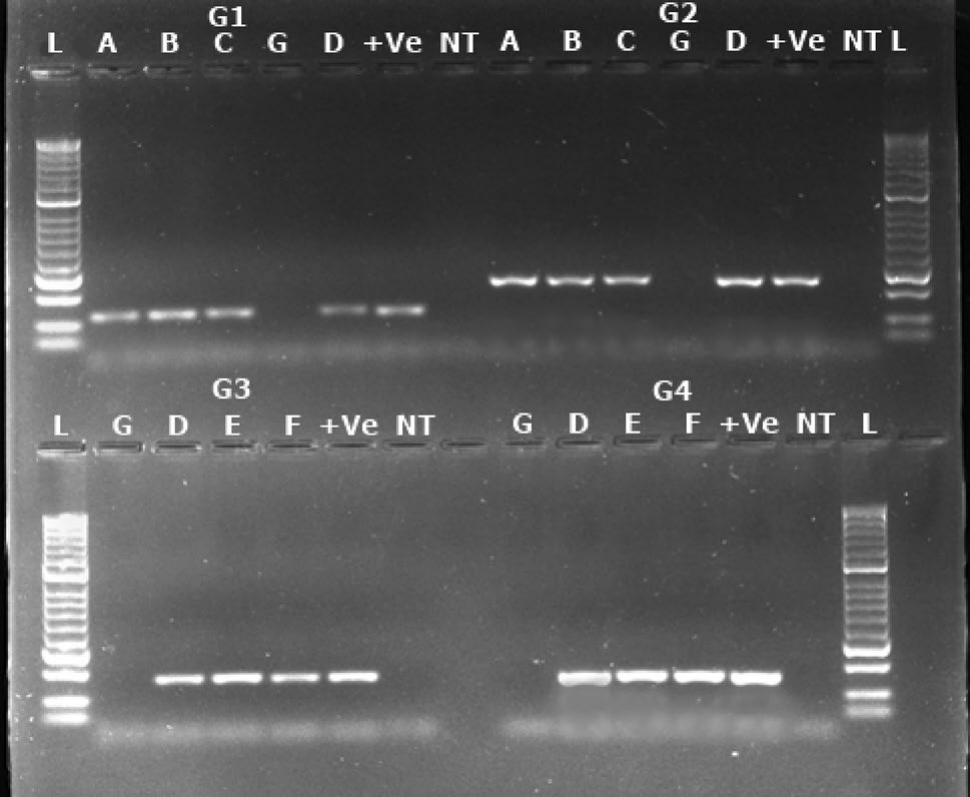
Amplifications for designed RPA primers for detection of cassava brown streak virus (CBSV) and Uganda cassava brown streak virus (UCBSV). G1 and G2 represent amplifications using RPA primers targeting the coat protein (CP) and cylindrical inclusion (CI) genes, respectively, for CBSV. G3 and G4 represent amplifications using primers targeting the CP and CI genes, respectively, for UCBSV. Lanes A, B and C: CBSV infected plant samples; lane D: Dual (CBSV and UCBSV) infected plant sample; Lanes E and F: UCBSV infected plant samples; lane G: Health control plant; lane NT: Non-template control; lane + ve: Dual (CBSV and UCBSV) infected positive plant sample; and L: 100 bp ladder (New England Biolabs).

Results showed that the best RPA primer set for CBSV detection was 8518-deg-F/8633-R, which amplified a 116 bp fragment of the CP gene, and for UCBSV detection, the best RPA primer set UCBSV-GSF2/UCBSV-GSR2 amplified a 163 bp fragment of the CI region. The results showed that the expected amplicons were obtained with these primers, producing strong, clear bands (Fig. 1). To confirm that the primers were specific for CBSV and UCBSV, the positive amplification products were purified and Sanger sequenced (Supplementary Table S2). BLASTn analysis showed a nucleotide identity of 96.30% and 99.13% for CBSV (GenBank accession number for GU563327.1) and UCBSV (GenBank accession number KR911728.1), respectively, confirming that the RPA primers were specific each target virus. Consequently, the primers sets 8518-deg-F/8633-R and UCBSV-GSF2/UCBSV-GSR2 for detection of CBSV and UCBSV, respectively, were selected for subsequent experiments.

### Real-time RT-RPA

**Figure 2:**
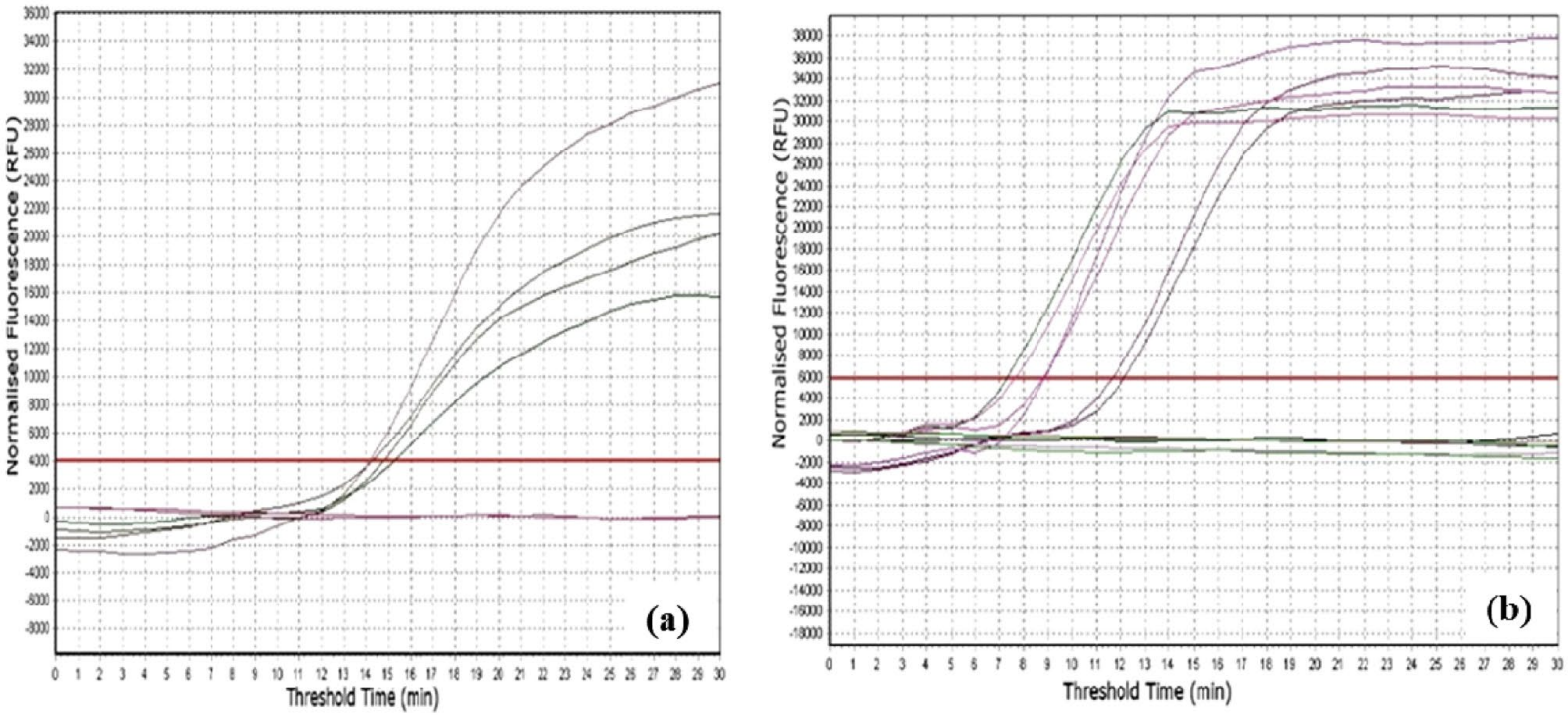
Amplification curves for real time RT-RPA assay for detection of cassava brown streak viruses. (**a**, **b**) represents amplification curves for detection of CBSV and UCBSV, respectively, as observed in Eppendorf’s Mastercycler ep realplex instrument.

After choosing the best RT-RPA primer set for CBSV and UCBSV detection, RPA exo probes were designed for each virus to enable the development of a real-time RT-RPA assay. The same RNAs obtained from the reference samples were tested by real-time RT-RPA and results were similar to those obtained by conventional RT-PCR using RPA primers, with positive amplification signals above the threshold obtained in less than 20 min (Fig. 2).

Crude extracts were prepared from the same leaves and stem samples used for RNA extraction. Real-time RT-RPA results using crude extracts as template agreed with those obtained with RNA as templates with similar amplification times.

### Comparisons between real time RT-RPA assay and conventional RT-PCR, RT-LAMP and qRT-PCR

The new real-time RT-RPA was further evaluated by testing additional nineteen cassava samples (symptomatic and asymptomatic for CBSD) obtained from the quarantine greenhouse collection at KEPHIS and results compared to real-time RT-LAMP, qRT-PCR and conventional RT-PCR (Table 2). An empirically derived scale was used to score the symptoms in a scale of 1–3 whereby 1 = no symptoms; 2 = mild symptoms and 3 = severe leaf symptoms (Supplementary Fig. S3). Four samples tested positive for UCBSV by all the four molecular diagnostic methods. For CBSV, there was one discrepancy between the methods as sample #8, tested positive by RT-LAMP and qRT-PCR and negative by real-time RT-RPA and conventional RT-PCR. Sample #17, showed no symptoms of CBSD infection but was detected as CBSV-positive by all the four diagnostic methods. All negative and positive controls in all the assays gave expected results with amplification observed in positive controls and no amplification in the negative and healthy controls.

**Table 2:** Comparison between real-time RPA, RT-PCR, RT-LAMP and qRT-PCR for detection of CBSV and UCBSV in cassava samples.

Sample #	Symptom scoring*	CBSV				UCBSV			
		RT-PCR	Real-time RT-RPA	RT-LAMP	RT-qPCR	RT-PCR	Real-time RPA	RT-LAMP	RT-qPCR
1	1	−	−	−	−	−	−	−	−
2	2	+	+	+	+	+	+	+	+
3	3	+	+	+	+	−	−	−	−
4	3	+	+	+	+	−	−	−	−
5	3	+	+	+	+	−	−	−	−
6	2	+	+	+	+	−	−	−	−
7	1	−	−	−	−	−	−	−	−
8	2	−	−	+	+	+	+	+	+
9	3	−	−	−	−	+	+	+	+
10	1	−	−	−	−	−	−	−	−
11	1	−	−	−	−	−	−	−	−
12	3	+	+	+	+	+	+	+	+
13	3	+	+	+	+	−	−	−	−
14	1	−	−	−	−	−	−	−	−
15	3	+	+	+	+	−	−	−	−
16	3	+	+	+	+	−	−	−	−
17	1	+	+	+	+	−	−	−	−
18	1	−	−	−	−	−	−	−	−
19	1	−	−	−	−	−	−	−	−

Symptom scoring severity scale: 1 = asymptomatic, 2 = mild symptoms, 3 = severe symptoms. (+) and (−) represent positive and negative, respectively, to either UCBSV or CBSV.

### Specificity and limit of detection of real time exo RT-RPA

**Figure 3:**
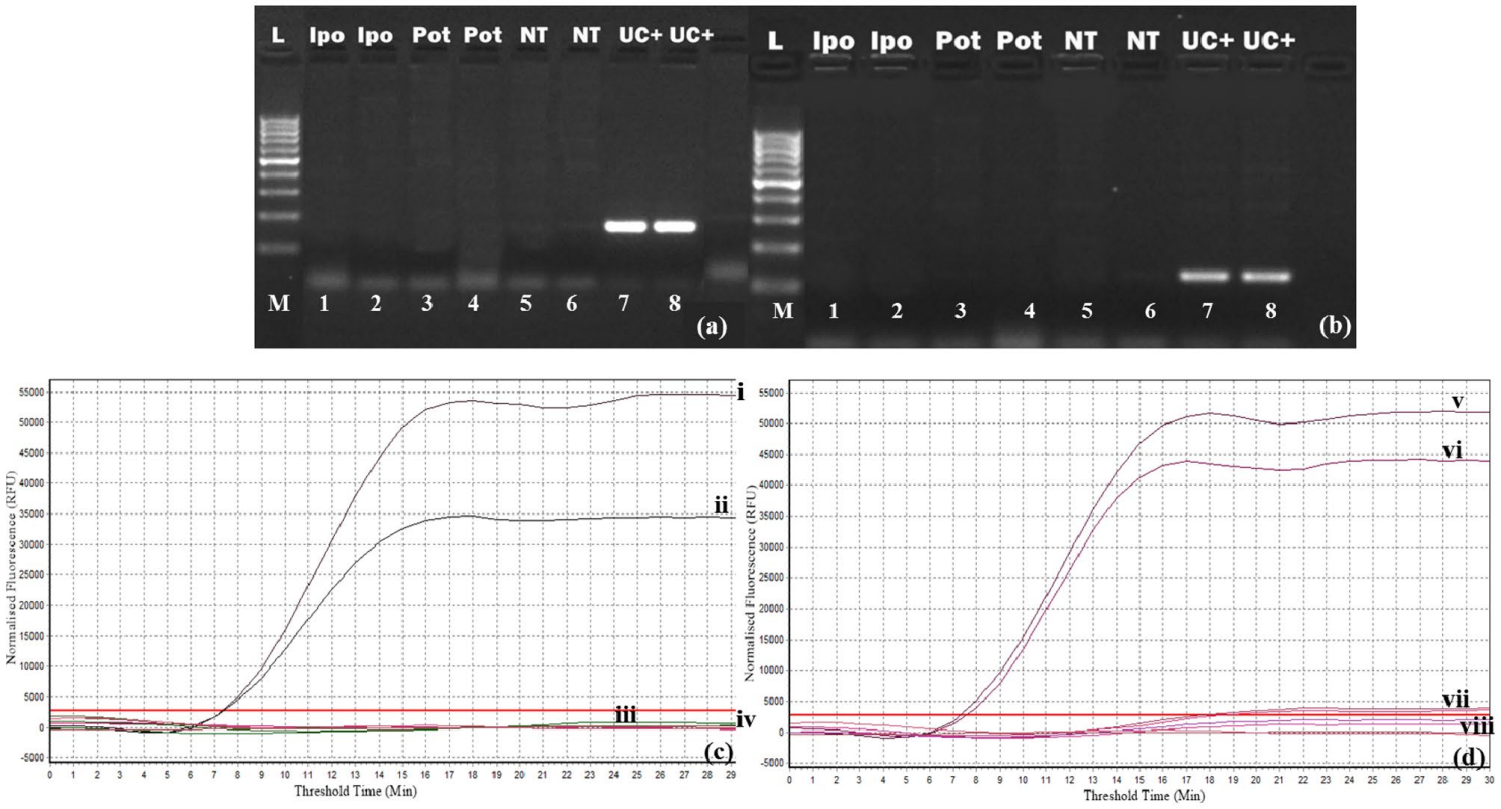
Specificity of RT-RPA assays for specific detection of cassava brown streak viruses by agarose gel electrophoresis and amplification curves. (**a**, **b**): Detection specificity of UCBSV- and CBSV-specific RT-RPA assay, respectively. Lane M, 100 bp ladder (New England Biolabs); Lanes 1 and 2, Sweet potato mild mottle virus (SPMMV), *Ipomovirus*; Lanes 3 and 4, Cowpea-aphid-born mosaic virus (CABMV), *Potyvirus*; Lanes 5 and 6, Healthy control; and Lanes 7 and 8, UC + (Dual [UCBSV and CBSV] infected positive control). (**c**) and (**d**): Real-time RT-RPA amplification curves for specificity using RPA primers for UCBSV and CBSV, respectively. Curves (i) and (ii) shows specific amplification for positive UCBSV samples; (v) and (vi) shows specific amplification for positive CBSV samples; (iii) and (vii), shows negative amplification for a sample infected with Sweet potato mild mottle virus (SPMMV), *Ipomovirus*; (iv) and (viii) shows negative amplification for a sample infected with Cowpea-aphid-born mosaic virus (CABMV), *Potyvirus*.

Specificity of the real-time RT-RPA assay was confirmed by cross reaction assays, i.e. testing CBSV and UCBSV primers and probes using UCBSV- and CBSV-positive samples, respectively. RNAs obtained from samples infected with Sweetpotato mild mottle virus (genus *Ipomovirus*) and Cowpea-aphid-born mosaic virus (genus *Potyvirus*) were also tested. No cross reactions occurred for any of the samples, indicating a high specificity of the primers used for each specific target (Fig. 3). Thus, the assay can be considered to be highly specific.

The detection limit of the real-time RT-RPA assay was determined by testing tenfold serial dilutions of purified RNAs obtained from CBSV or UCBSV-infected stem and leaf samples and by comparing results with conventional RT-PCR RT-LAMP and qRT-PCR. Ugandan cassava brown streak virus (UCBSV*)* was detected consistently down to the 1 × 10^–5^ dilution using RNA obtained from either stem or leaf samples (Table 3). This sensitivity is equivalent to the one obtained by qRT-PCR and 10 × higher than RT-LAMP when testing stem samples. For CBSV (Table 4), consistent positive results were obtained down to the 1 × 10^–5^ dilution, a sensitivity similar to the one obtained by RT-LAMP and 10 × higher than qRT-PCR for cassava leaf samples. On the other hand, when testing stem samples, no positive results were obtained at dilutions lower than 1 × 10^–3^. Similar results were obtained from qRT-PCR and RT-LAMP.

**Table 3:** UCBSV leaf and stem serial dilution results showing comparisons of RT-RPA with other molecular diagnostic methods; *FL4L* leaf sample, *FL4S* stem sample, (+) positive, (−) negative.

Sample/dilutions	Sample type	10^0^	10^–1^	10^–2^	10^–3^	10^–4^	10^–5^
FL4L-UCBSV	cDNA-LAMP	++	++	++	++	++	++
	cDNA-RPA	++	++	++	++	++	++
	cDNA-qPCR	++	++	++	++	++	++
	cDNA-PCR	++	++	–	–	–	–
FL4S -UCBSV	cDNA-LAMP	++	++	++	++	++	–
	cDNA-RPA	++	++	++	++	++	++
	cDNA-qPCR	++	++	++	++	++	++
	cDNA-PCR	++	++	–	–	–	–

**Table 4:** CBSV serial dilution series from stem and leaf sample and cDNA and comparisons of RT-RPA with other molecular diagnostic methods; *MF3L* leaf sample, *MF3S* stem sample, ( +) positive, (−) negative.

Sample/dilutions	Sample type	10^0^	10^–1^	10^–2^	10^–3^	10^–4^	10^–5^
MF3L-CBSV	cDNA-qPCR	++	++	++	++	++	−−
	cDNA-LAMP	++	++	++	++	++	++
	cDNA-RPA	++	++	++	++	++	++
	cDNA-PCR	++	++	++	−−	−−	−−
MF3S-CBSV	cDNA-qPCR	++	++	++	++	−−	−−
	cDNA-LAMP	++	++	++	++	−−	−−
	cDNA-RPA	++	++	++	++	−−	−−
	cDNA-PCR	++	++	−−	−−	−−	−−

## Discussion

Cassava brown streak disease is a threat to food security in sub-Saharan Africa^1^. The brown corky necrotic lesions caused by the disease can spread throughout the entire starch-rich storage roots rendering them inedible and leading to food shortages and high economic impact to smallholder farmers^4^. Cassava plants infected with cassava brown streak viruses might not express any symptoms of the disease (as was the case of sample #17 in this work). Further, variations in symptoms development can be related to several factors including virus species, cassava cultivar and plant age and disease symptoms can be confused with the natural leaf senescence. This highlights the importance of early detection of the disease because by missing the symptoms and/or aboveground plants are asymptomatic farmers will not be aware of the disease until harvest time. The development and availability of field-based diagnostics are important to support early disease detection.

The qRT-RPA test developed in this study showed high specificity and sensitivity in detecting CBSV and UCBSV in both symptomatic and asymptomatic cassava plants. This test has the potential to be used as an on-site test to detect CBSD in farmers’ cassava fields early in the growing season at a time when farmers can rogue eradicate infected plants and replant using virus-free stem cuttings. The early detection of the disease will also prevent its spread to neighboring plants by the whitefly vector *Bemisia tabaci*^43^. The RT-RPA test can also support seed certification schemes to guarantee the production of virus-free planting materials for bulking, the work of cassava breeders in selecting for resistance to CBSD and cross-border trade inspections on cassava planting materials.

The RPA primers and probes were designed based on the CI and CP regions of UCBSV and CBSV genomes, respectively. The reason for targeting CI region and not only the CP region where most sequencing information is available is that previous studies^44–46^ have reported CI as the most genetically stable region especially in potyviruses and therefore a better target for design primers for diagnostic assays. In our study, the evaluation of RPA primers showed that this was true for UCBSV but for CBSV the primers designed for the CP region gave the best results. The qRT-RPA test was compared to other available methods to detect CBSV and UCBSV. The RT-RPA was shown to be as sensitive or 10 times more sensitive than LAMP and qRT-PCR and with high specificity as no cross reactions with other potyviruses were detected.

Results of the present study demonstrated that the crude plant extracts can be used directly as templates for reliable detection of CBSV and UCBSV by the RT-RPA test in under 15 min. The use of crude extracts offers several benefits as it avoids the laborious process of RNA extractions, reducing the costs and processing time and facilitates the adoption of the test in low resources settings. Further, in the context of seed production systems, crude extracts are highly suitable for large scale testing due to its simplicity and minimal equipment requirements^40^. Though the crude plant extracts in this study were done using PEG buffer, there are other available extraction buffers (e.g., 0.5 M NaoH solution^39^, phosphate buffer PH 7.4, NaoH: EDTA (1:1)^47^ that could be explored in future to further make the assay affordable based on the available buffers in the laboratories.

The real-time detection of the RT-RPA products can be done using a laboratory real-time PCR machine or low-cost instruments such as the Genie® II from OptiGene, reducing the risk of contamination associated with post-amplification processes (e.g. gel electrophoresis). The use of portable and battery-powered instruments to visualize RT-RPA reactions facilitates the adoption of the assay not only as an on-site test but also in laboratories with less resources to acquire expensive machinery and/or experiencing repeated power failures.

In this study, the same fluorophore (FAM) was used in both CBSV and UCBSV probes. Initially the CBSV probe was labelled with HEX fluorophore. However, due to the real-time PCR machine used in this study not having the HEX channel this prompted re-synthesis of the CBSV probe with FAM fluorophore. The use of two probes labelled with different fluorophores would allow duplex reactions, where both targets (CBSV and UCBSV) can be detected in a single reaction, to be performed. This would further reduce the cost, time and labor input associated with simplex assays. However, considerations need to be made on the choice of reporter and quencher dyes in terms of the wavelength and fluorescence intensity as well as fluorophore-machine compatibility^48,49^.

Although CBSD was confined to coastal eastern Africa for several decades, it is currently a major problem affecting cassava productivity in East, Southern and Central Africa. The existence of CBSD in Democratic Republic of Congo (DRC)^50,51^ highlights the high risk of CBSD spreading to West Africa, posing a major threat to the important cassava-producing countries of West Africa. Monitoring the spread of CBSD to West Africa is critical, since cassava is the most important staple food in West Africa, particularly Nigeria, which is the world’s largest cassava producer. The increased threat of spread of CBSD westwards of Africa calls for adoption of improved molecular diagnostic assays such as RT-RPA to monitor and track the disease spread and increase the level of preparedness and response. Therefore, the developed assay can be adopted by programs such as West African virus epidemiology (WAVE, https://wave-center.org/) and used across the partner countries for improved early and real-time detection of CBSD. The developed RT-RPA will contribute to an improved diagnosis of CBSD and strengthen the testing capability of National Agricultural Research Systems (NARS) for routine screening of cassava germplasm, virus indexing on a large scale, certifying planting materials and breeding for virus resistance.

## Conclusion

Improved virus detection plays a significant role in disease prevention and management. The present study developed a rapid, sensitive and specific assay based on recombinase polymerase amplification for detection of CBSV and UCBSV. The direct RPA assay using crude plant extract overcomes the challenges and cost associated with the RNA extraction step, ensuring results are obtained in less than 20 min with high specificity and sensitivity. Due to the ease of sample preparation as crude extracts and the use of low temperature, there is need to test the developed diagnostic assay for on-site field detection of cassava brown streak viruses so that the method can benefit smallholder farmers as well nurseries and under-equipped laboratories in low resource settings.

This study is the first report for detection of CBSVs directly from crude extract samples obtained from cassava leaf and stem tissues. There is a need to establish duplex/multiplex assays for simultaneous detection of CBSV and UCBSV in a single reaction. This could further contribute to reducing the time used in the detection of the viruses for routine diagnostics. Successful implementation of the assay will strengthen the efforts to provide clean cassava planting materials preventing disease spread and outbreaks.

## Materials and methods

### Cassava plant materials

**Figure 4:**
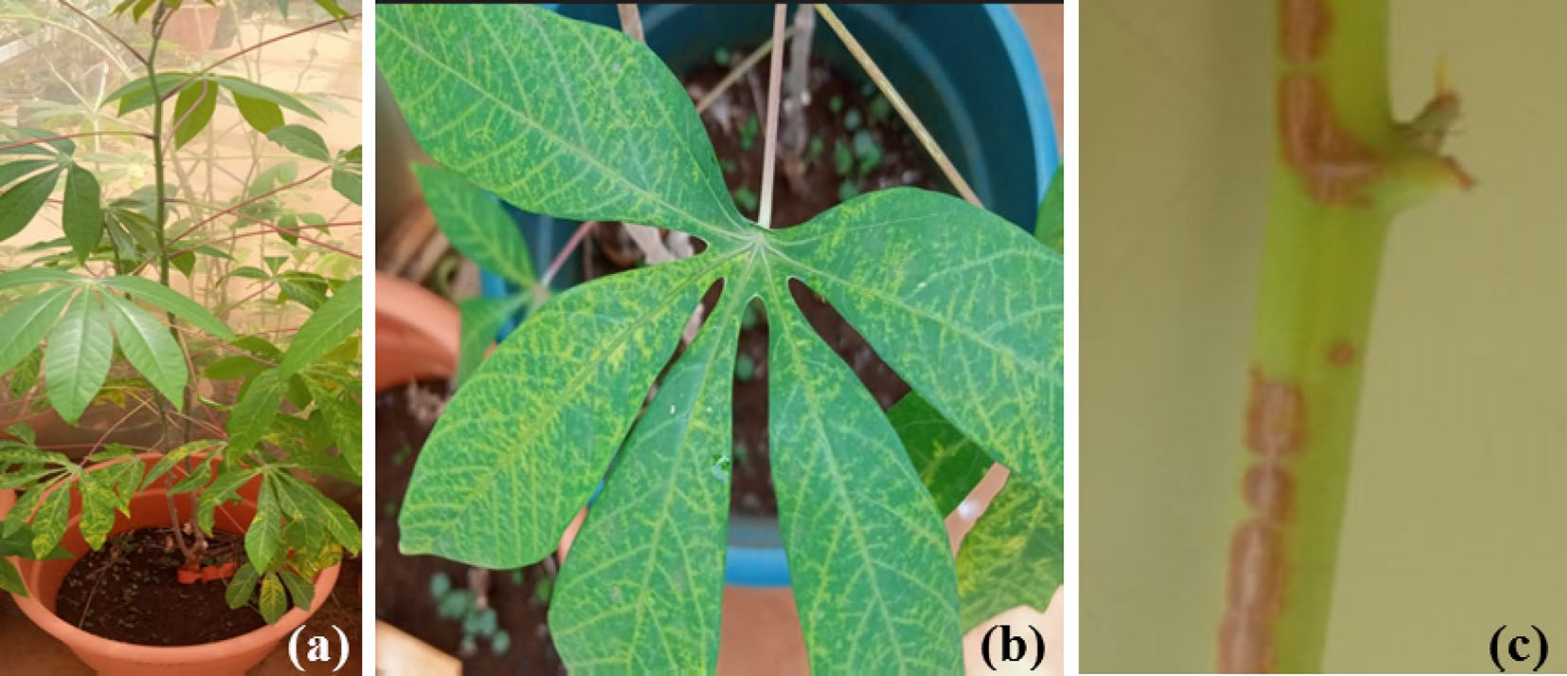
Cassava leaf and stem samples collected from the quarantine greenhouse showing characteristic CBSD symptoms. (**a**, **b**) feathery chlorosis and yellowing along the leaf veins; and (**c**) a young cassava stem with brown lesion symptoms.

Positive and negative reference plant samples used in the present study were obtained from a collection of virus-infected cassava plants maintained in an insect-proof greenhouse at Plant Quarantine & Biosecurity station of Kenya Plant Health Inspectorate Service (KEPHIS), Kenya following approval to access the materials maintained by the institution. Six plant samples showing various CBSD characteristic symptoms on the leaves including feathery chlorosis, chlorotic blotches, yellowing along the veins (Fig. 4a,b) and brown lesions on the stems (Fig. 4c) were selected and used for the experiments. The presence of CBSV and UCBSV in the selected plant samples was confirmed using conventional RT-PCR with primers targeting the coat protein gene as described by Winter et al.^9^. Cassava plantlets from the tissue culture laboratory, previously confirmed negative for the two viruses were used as healthy controls. Additional leaf samples from asymptomatic and symptomatic cassava plants (n = 19) were used for further evaluations and comparisons of the developed RT-RPA assays with other methods to assess the RT-RPA performance.

### Total RNA extraction and preparation of crude extract samples

Total RNA was isolated from the cassava leave samples using the RNeasy Plant Mini kit (QIAGEN, Valencia, CA) following manufacture’s protocol. The quality and concentration of the extracted RNAs were assessed using Nanodrop™One spectrophotometer (Thermo Scientific, Wilmington, USA). The protocol for the detection of CBSV and UCBSV from crude extracts was adapted from Silva et al.^40^. For cassava leaf samples, round discs were obtained using a sterilized 1 cm glass tube. The parts of the stem showing CBSD symptoms were scrapped off using a sterile scalpel blade. The leaf discs and the stem scrapping were immersed in 300 µl of a freshly prepared polyethylene glycol (PEG) buffer (6% PEG-200 with 20 mM NaOH). The tubes were vortexed briefly and incubated for 5 min at room temperature. Crude extracts were used directly as templates in RT-RPA assays.

### RPA primer and exo probe design and synthesis

A multiple http://www.ncbi.nlm.nih.gov/genbank/ sequence alignment of 80 CBSV and UCBSV reference nucleotide sequences obtained from the NCBI GenBank database was performed in Geneious Prime® 2021.1.1 (Biomatters Ltd., Auckland, New Zealand). The design of RPA primers (Table 5) and exo probes (Table 6) was done following TwistDx (www.twistdx.co.uk) recommendations. Two sets of primers were designed targeting the coat protein (CP) and the cylindrical inclusion (CI) regions of CBSV and UCBSV genomes. RPA exo probes were designed after identifying the best primer set for CBSV and UCBSV. The designed probes contained a tetrahydrofuran residue (THF) which replaces a nucleotide in the target sequence, flanked by a dT-FAM-fluorophore (thymine labelled with 6-carboxyfluorescein and a dT-BHQ1 (thymine labelled with Black Hole quencher group, which quenches FAM in close proximity. The probes also contained a 3ʹ end modification group (C3 spacer) blocking any polymerase mediated extension as per the instructions in the primer design manual*.* The RPA primers and probes were synthesized at Eurogentec S.A. (Seraing, Belgium).

**Table 5:** RPA primers designed in this study for UCBSV and CBSV detection.

Primer name	Sequence (5ʹ–3ʹ)	Target virus	Product size (bp)	Region*
GSF2	CCTTTATTCTCATTGTTGCGTGAGTTGAGCCC	UCBSV	163	Cylindrical inclusion (CI)
GSR2	ATGTTCCTTGCAATTCAGCCCATTTCTTGAC			
8518-F	GGATTGTCAATTGTATTGTGAATGGGACTA	CBSV	116	Coat protein (CP)
8633-R	CATAGGGTATTCTGAATCATCAATATCCTC

**Table 6:** RPA probes for UCBSV and CBSV, 1 = dT-FAM; 2 = BHQ2-dT; Z = THF.

Probe name	Sequence (5ʹ–3ʹ)
UCBSV-RPA-probe	AYGTTGATTTATCAACTAATCAYAAAGT1GZ2ATACAYACRCTTGA-Spacer C3
CBSV-RPA-probe	GARAGGAAGAATGAGAAATCGTGGAGAGC1Z2TGAGYTAAATGCRCA-Spacer C3

### Selection of RPA primers for CBSV and UCBSV detection using RT-PCR assay

The RPA primer sets designed in this study were initially evaluated using a conventional RT-PCR. Total RNAs from known negative and positive samples for CBSV and UCBSV maintained in a greenhouse collection were used as templates. Reverse transcription was carried out in 20 µl reactions containing 2 µl total RNA, 1 × M-MLV reaction buffer (NEB, USA), 0.5 mM each dNTP, 0.5 µM oligo (dT) primer, 0.5 µM random N primer and 200 units of Moloney Murine Leukemia Virus (M-MLV) reverse transcriptase (NEB, USA). A mixture of RNA and primer was heated to 70 °C for 5 min then placed on ice, after which the remaining components were added and the reactions incubated at 25 °C for 5 min, 42 °C for 60 min and finally at 80 °C for 3 min. The RT-PCR assay was performed using 2 × master mix (Jena Bioscience) in a 25 µl reaction volume in an Applied Biosystems 9700 thermocycler (Applied Biosystems, Foster City, CA, USA). The thermal cycling conditions used were as follows; 4 min at 95 °C followed by 35 cycles of 30 s at 95 °C, 40 s at 60 °C and 45 s at 72 °C and final extension at 72 °C for 10 min. The PCR products were separated in 1.5% agarose gel stained with Gel red (Biotium Hayward, CA USA). The images were captured using C200 gel doc (Azure Biosytems). Based on the presence of clear and distinct bands of the expected sizes of 176 bp and 493 bp for UCBSV and CBSV, respectively, the best primer pairs for CBSV and UCBSV detection were selected and used to determine the best region for design of the probes for subsequent development of the RPA assay.

### Optimization of real-time RT-RPA assay

Complementary DNA (cDNA) synthesis was carried out in 20 µl reactions containing 2 µl of total RNA, 1 × M-MLV reaction buffer (New England Biolabs, USA), 0.5 mM each dNTP, 0.5 µM oligo (dT) primer, 0.5 µM random N primer and 200 units M-MLV reverse transcriptase (New England Biolabs, USA). A mixture of total RNA and primers was heated to 70 °C for 5 min then placed on ice, after which the remaining components were added and the reactions incubated at 25 °C for 5 min, 42 °C for 60 min and a final incubation at 80 °C for 3 min.

The real time RT-RPA reactions were carried out with the Twist-Amp exo kit (TwistDx Ltd; Cambridge, UK) following manufacturer’s guidelines. The RPA reactions were performed in 12.5 µl reaction volume containing 420 nM of both CBSV and UCBSV RPA primers, 120 nM of each RPA exo probe for the two viruses, 14 mM magnesium acetate and TwistAmp rehydration buffer. All the reagents except for magnesium acetate and the template were prepared in a mastermix, which was used to rehydrate the freeze-dried enzyme pellets. The solution was then transferred into 0.2 ml PCR tubes and 2 µl of cDNA was added. The reactions were initiated by adding the magnesium acetate into the cap of each tube, recapped, centrifuged briefly and immediately placed into a real-time PCR instrument (Eppendorf’s Mastercycler realplex2, Eppendorf, Cambridge, UK). The fluorescence data was measured at 37 °C every 60 s using FAM channel. For one-step RT-RPA, 0.5 U of M-MuLV Reverse Transcriptase (New England Biolabs, UK) and 6 U of RNase Inhibitor (NEB, UK) were added to the mastermix described above and 2 µL of purified RNA was used as template in 12.5 µL reactions. All the samples were run in duplicates, a positive and a negative control was included in the assay. Primers targeting plant cytochrome oxidase gene (COX)^34^ were used as an internal control to confirm quality the templates used in the RPA reactions.

### Direct RT-RPA for detection of CBSV and UCBSV in crude plant extract

The same leaf and stem samples used for RNA extraction were used to prepare crude plant extracts for the detection of CBSV and UCBSV. The crude extracts and the real time exo RT-RPA reactions were set up as described above and using 2 µl of crude extract as templates. Each sample was tested in duplicate and repeated twice. Positive and negative controls were included in the tests.

### Specificity and limit of detection of RT-RPA assay in detection of CBSV and UCBSV

To test for the specificity of the real-time RT-RPA for detection of the CBSV and UCBSV, samples that were known to be positive for UCBSV were tested with CBSV primers and vice-versa. Samples infected with Sweet potato mild mottle virus (SPMMV; genus *Ipomovirus*) and Cowpea aphid-borne mosaic virus (CABMV; genus *Potyvirus*) were also included in the assay. The tests for all the samples were done in duplicate and repeated twice. The limit of detection for the developed RT-RPA assay for both CBSV and UCBSV using the primers 8518-deg-F/8633-R and UCBSV-GSF2/UCBSV-GSR2, respectively, was determined using tenfold serial dilutions of crude extracts, cDNAs and RNAs obtained from infected leaf and stem samples. The limit of detection of the real time exo RT-RPA was compared with conventional RT-PCR, RT-LAMP and qRT-PCR.

### Evaluation of the real-time RT-RPA assay and comparison with RT-LAMP, conventional RT-PCR and qPCR for CBSV and UCBSV detection

To evaluate the developed assay, nineteen RNAs obtained from leaf samples collected from symptomatic and asymptomatic cassava plants were tested with RT-RPA and results compared with conventional RT-PCR^9^, qRT-PCR^18^ and real-time RT-LAMP^34^. The oligonucleotide primers used for RT-PCR, qRT-PCR and real-time RT-LAMP are presented in Supplementary Table S1.

RT-RPA was carried out as described in the previous sections. For conventional RT-PCR carried out as described by Winter et al.^9^, a 25 µl reaction volume was performed using 2 × master mix (Jena Bioscience) with thermal cycling conditions as follows: 4 min at 95 °C followed by 35 cycles of 30 s at 95 °C, 40 s at 60 °C and 45 s at 72 °C and final extension at 72 °C for 10 min. Amplification products were analyzed using 1.5% agarose gel electrophoresis. The resultant images were captured using C200 gel doc (Azure Biosystems). Quantitative real time reverse transcription PCR (qRT-PCR) reactions were set up as 20 µl reactions containing 2 × master mix (Jena Bioscience), 300 nM of each primer and 100 nM of CBSV and UCBSV probes as described by Adams et al.^18^. The targets were amplified using the Eppendorf’s Mastercycler ep realplex (Eppendorf, Cambridge, UK) in a cycle consisting of 2 min at 95 °C followed by 40 cycles of 15 s at 95 °C and 60 s at 60 °C in a single run. Real-time RT-LAMP was performed for CBSV and UCBSV in a Genie II instrument (OptiGene Limited, West Sussex, UK) as described by Tomlinson et al.^34^. A 15 µl reactions containing 7.5 µl Isothermal Master Mix (OptiGene), 200 nM each external primer (F3 and B3), 2 µM each internal primer (FIP and BIP), 1 µM each loop primer (F-loop and B-loop), 1.2 units M-MuLV Reverse Transcriptase (New England Biolabs, USA) was carried out. Reactions were incubated at 65 °C for 30 min and then subjected to a slow annealing step (0.05℃/s) from 95 to 75 °C with fluorescence monitoring. Reactions containing water instead of RNA were included in each run as a non-template control. All the assays were performed in duplicates including both positive and negative controls.

### Sources of experimental plant material

All plants used in the present study belong to Plant Quarantine and Biosecurity Station, Muguga, at Kenya Plant Health Inspectorate service.

### Ethics statement

Ethical approval was not required for this study. The authors confirm that the experiments that used plants and their parts in the present study comply with International, National and Institutional Guidelines.

### Supplementary Information

Supplementary Information.

## Data Availability

The data that supports the finding of this study are included in this article and its supplementary material. The sequences of the amplicons to confirm the specificity of the primers can be found in the supplementary material. Further inquiries on raw data can be availed by the corresponding author upon request.
