# Supplementary material for: Real-time reverse transcription recombinase polymerase amplification (RT-RPA) assay for detection of cassava brown streak viruses
Source: Sci Rep. 2024 May 30;14:12438. doi: 10.1038/s41598-024-62249-y (PMC11139904; doi:10.1038/s41598-024-62249-y)
Supplement: Supplementary file 1 — Supplementary Information. [file 41598_2024_62249_MOESM1_ESM.docx]

Supplementary Material

Real-time reverse transcription recombinase polymerase amplification (RT-RPA) assay for detection of cassava brown streak viruses

**Florence Munguti^1, 2*^, Dora C. Kilalo^1^, Hillary K. Yegon^2^, Isaac Macharia^2^, Susan Seal^3^, Agnes W. Mwango’mbe^1^, Evans N. Nyaboga^4^, Gonçalo Silva^3^**

^1^Department of Plant Science and Crop Protection, University Nairobi, Kenya

^2^Kenya Plant Health Inspectorate Service, Nairobi, Kenya

^3^Natural Resources Institute, University of Greenwich, Central Avenue, Chatham Maritime, ME4 4TB, United Kingdom

^4^Department of Biochemistry, University of Nairobi, Kenya

***Corresponding author**

Florence Munguti

Kenya Plant Health Inspectorate Service, Nairobi, Kenya

Email address: fmunguti@kephis.org

**Supplementary Tables**

**Supplementary Table S1.** RT-qPCR, LAMP and RT-PCR primers used in the study.

| **Test** | **Primer name** | **Sequence 5’-3’** | **Reference** |
| --- | --- | --- | --- |
| **RT-PCR** | UCBSV gen F | AACAGACATACGTGTGCAT | Winter et al.^9^ |
|  | UCBSV gen R | ATTTCCAGGTTCCTTTGTCACT |  |
|  | CBSV gen F | GTACGTGCCTCCATCACAT |  |
|  | CBSV gen R | CTCAACAGCTCTCCACGATTT |  |
| **qRT-PCR** | UCBSV forward | GATYAARAAGACITTCAAGCCTCCAAA | Adams *et al*.^18^ |
|  | UCBSV reverse | AATTACATCAGGRGTTAGRTTRTCCCTT |  |
|  | UCBSV probe | FAM-TCAGCTTACATTTGGATTCCACGCTCTCA- TAMRA |  |
|  | CBSV forward | GCCAACTARAACTCGAAGTCCATT |  |
|  | CBSV reverse | TTCAGTTGTTTAAGCAGTTCGTTCA |  |
|  | CBSV probe | FAM- AGTCAAGGAGGCTTCGTGCYCCTC -BHQ1 |  |
| **LAMP** | CBSV F3 | CGACRATGAGGAAAATAATGAGAAAT | Tomlinson *et al*.^34^ |
|  | CBSV B3 | GAACAACTTRGTTTTATTTCTACCAA |  |
|  | CBSV FIP | TTTTCAATGCTTGTATACCCAGCACGATCAGAATAGTGTGWCTGCTGGA |  |
|  | CBSV BIP | GGTATTGACTTCCTAGCCGAAGCATTAGCAGCCAGTATTTGATGTTT |  |
|  | CBSV F-Loop1 | TTCGGGCTGCTTTTATYACAA |  |
|  | CBSV B-Loop2 | ACAAYTGTCACAAAGCCAACT |  |
|  | UCBSV F3 | AATYCCAACWARTGCTCTTGAGAT |  |
|  | UCBSV B3 | TATTAACTCCATATGCTTTAGCAAC |  |
|  | UCBSV FIP | CCTTTGAGAGCGYGGAATCAAGACDTTCAAGCCTCCAAA |  |
|  | UCBSV BIP | TTTCCTGGCATAYRTACCTCCATTGCCCAATTYTCAACTTCAA |  |
|  | UCBSV F-Loop3 | CAAATGTAAGCTGACTGTGAYAC |  |
|  | UCBSV B-Loop2 | CTCAYGCTATAGATAAYCAACTTGC |  |

**Supplementary Table S2.** Sequences of the amplicons to confirm the specificity of the primers for CBSV and UCBSV

| **Target virus** | **Sequence** |
| --- | --- |
| CBSV | >200409-R01_I07_L1_CBSV-B3.ab1 604  GAGCATATTTGAACGTTCGGAAACCAGTTGGCTATGTGATAATTGTGCTT  CGGCTAGGAAGTCAATACCCAACCATTTATTTTCAATACTTGTATACCCA  GCATTTCGGGCCGCTTTTATTACAAAAGCTTTTCCAGCAGTCACACTATT  CTGATACATTAAAATTGCTTGGCTGGAAAAATTTCTCATTATTTCCCTCC  ATCCGTCGAAAAGGGGGGGGTTGGGGGCACCCCCCCCCCCCCCCCCACCC  CCCCAACCCCCCCAAAACCACCCCCAAAAAAAAAAAAAAAAAAAAAAATA  AAAGATACTCAAAATACACAAGAGAAAAAAAAAAAAAAAAAAAAAAAAAA  AAAAAAAAAAAAAAAAAAAAAAAAAAAAAAAAAAAAAAAAAAAAAAAAAA  AAAAAAAAAAAAAAAAAAAAAAAAAAAAAAAAAAAAAAAAAAAAAAAAAA  AAAAAAAAAAAAAGAAAAAAAAAAAAAAAATTGGGAATGATAAAAAAAAA  AAAAACATACTAAAAAAAAAAAAAAAAAAAAAAAAAAAAATTTTTGAAGA  AAAAGGCTGAAAAAATGAAAAAAGATAAACTTAAGCTAAAAAAAAAAAAA  ACTA |
| UCBSV | GGTGTATATCAACTTTGTGGTTGGTTGATAAATCAACATTGTGGCCAACA  TGTGTTGCTGATGTCTTGATTATTTTCTTGTCAGGGCTCAACTCACGCAA  CAATGAGAATAAAGGAGGCTGCTATGTGGGGTTTTTTGAGGGTTTGAAAG  ATTGGGCGTGGCTGAAGTCTTGCGTTGTTCGTACGATCGTCGATAGGTTA  CTTAATTTTTTTCTTATTAATGCGACGCGCAGCAGAGCGGCGACGGGCGG  AGGCAGGTTCTTTGCTTCTTTCCTTTGCTTGATTTCTTCCGTATTTTTCG  GAGCTTGGCTCCCTCGGTCGGTTGTGTACGTCAATGCGCTCGTTGTTTCT  GGTTTCCCCTTTATTTTTTCGGTCTTCCTCTCGCTTTTTGGGAGCCTTCG  GGGAGGGGTGTTTTGTCTTTTTTTGGGTGTGTGTTTCGGCCAGGGGTAGG  TCCCCTTCCCCCTTTTTCCCCTTTCCCCCCGCCTCCCGGGCGTGGTTTTT  TTTTTCAGGGAAGTTGGTGTGTAGACGTTGCTCTTTTTTTTTTCCCTTAT  CTTGCTTGGTGGTTCCCTAAATTTTATGGCTGTACCGCATATTTTTGGTG  >200409-R01_I05_R8_UCBSV-GSR2.ab1 2093  GGTGTATATCAACTTTGTGGTTGGTTGATAAATCAACATTGTGGCCAACA  TGTGTTGCTGATGTCTTGATTATTTTCTTGTCAGGGCTCAACTCACGCAA  CAATGAGAATAAAGGAGGCTGCTATGTGGGGTTTTTTGAGGGTTTGAAAG  ATTGGGCGTGGCTGAAGTCTTGCGTTGTTCGTACGATCGTCGATAGGTTA  CTTAATTTTTTTCTTATTAATGCGACGCGCAGCAGAGCGGCGACGGGCGG  AGGCAGGTTCTTTGCTTCTTTCCTTTGCTTGATTTCTTCCGTATTTTTCG  GAGCTTGGCTCCCTCGGTCGGTTGTGTACGTCAATGCGCTCGTTGTTTCT  GGTTTCCCCTTTATTTTTTCGGTCTTCCTCTCGCTTTTTGGGAGCCTTCG  GGGAGGGGTGTTTTGTCTTTTTTTGGGTGTGTGTTTCGGCCAGGGGTAGG  TCCCCTTCCCCCTTTTTCCCCTTTCCCCCCGCCTCCCGGGCGTGGTTTTT  TTTTTCAGGGAAGTTGGTGTGTAGACGTTGCTCTTTTTTTTTTCCCTTAT  CTTGCTTGGTGGTTCCCTAAATTTTATGGCTGTACCGCATATTTTTGGTG  GGGTCTTCTTTTCTTCCTTTTTTTGCCTTTTTTGTTAAGCTGGGGAGAGG  GGCGGTTTGGCCATGATTTTTTGTTTGAGTTTTAGTTTTTTTACGTTTCG  GGGTTGGGGTTTTGCCTTTCCCCTTTTTTTCCCTTTTCTTTCTCCGTTGG  GGTGGGGTTGCGTTTGCCCTTTATTGTTTTTCCAACTTTTCCCTCCAAAA  TTTCTCCGGGTTGGGGTGTTTCCCTGTTTTTTTGGGTTGCCCCCTTTTCA  CCATTATGTTTTATTCCTTGTTTTGGGCAAGGAATTGTTGTTTGTTTTTT  TTTCGCTTGAGCTTGGGGGCCTTTGGTTTTATAACCCGGGTCCGGTGTGG  CCTTTTTTTTTTGTTACTTTTTTTGCTTTTCTCGTTGAGGCCCAGTGTTC  CTTCGGGGTATGGTGGGGGCTGTGTGGGATTTCCCTCCCTACCCCAGGGT  ATGGCTTCTTTTTTGTATGGTTCGTTCTTTTGTTTGGTAGGGTCGTGGGG  CTGTTTTGCCCCGTTATTTCTTTTTTTGTTGGGCCGTGGGAGGGTGCTTT  GTTGTTTTTGTTTTTTTTTTTTTTTTTTTTTGCGGGTTGGCCTTTTCCCG  GGGGCGCCTGTGTGTTTTATTTTGAGGGGATTCCCTTTTAATTTCTTTTA  CGGGTTTTCTCTCTGTCCTTCTTGTGTTTGGTTCTTTGGTTGTTGCGAAT  GATAGAGTTATTAATCTTTATACTTTTTTTGTTTTGGTTTCTGTTTTTGC  GTTTGGTTTATTTGCTATGTTTTTGAGTTGTGCTGTGCTTTCTTTGTTTT  TTTTTTTGGCGTGTATATTCTTACCTGCTTGCGCGTGTTTGTTTGTTTGT  TTGTATTTTATGCCCTTGCGGTGGCTGCGTGCTCTTTCGTTTACTTGCAT  TTTTTGTGTGCTCTTGTGGTCTTTTTTTAGATGTGGCTCGTTTCTTTGGT  GTTGTTCTTTCTTTGAGTTATTGGGTTTTTTTATGTTTCACGTGTGCATG  TATTGTTGCTTTGTTTTTTTTGTTTGGTACTACTTCGCTTTTTTTTTGTA  CGGCTCTGATTTGCTGTTCTTGCGTTTGGATCTTTTGTTGGTTTTTTGTT  GTACATTTTTGCTGCCATTTTGCGTTGCGTGTTTTTTTTTCTTTGTCTGT  TGTGTTGTTTTTTTTGTCGTTTATCTGCTATGTTTTATGTCTCTCTGTGT  TTTTTTCTTGATGTGTCTGCTTTTTGGTGTTCCTTTGTTTCTGTTTCTGA  TGTTGTTCTTTTGTGAGTTTTGTGCTTTTTTTGTGGGTTTGGTTCTATTG  TAGTGTTTGTGCTGGTTTTTTTTGAGGTTATTTGTGTGATTTTATGTGGG  TTTATTTTTTGCTTTTTTGTTTGTTTTTTCATTGTGTGTTCTTTGGTGGT  TTTTTATTTTTTCTCCTGGTTTGTTTTGATTGTCTGTGTATTTATTTTTT  TTTCTACTCCAATTGGTTGATGTGACTATTTTTTGGGCGTTTT |

**Supplementary Figures**

**
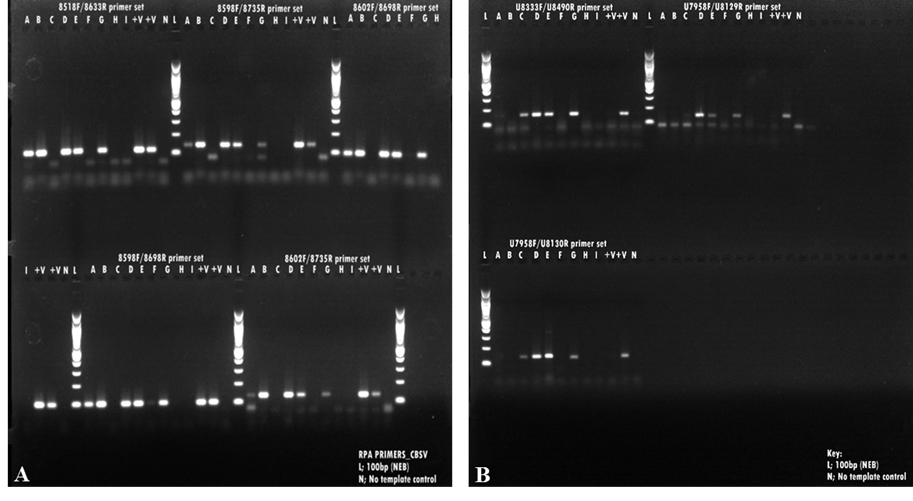
**

**Supplementary Figure S1: A and B** shows a gel indicating the screening of designed RPA primers targeting the coat protein (CP) region. Lanes A, B and C: CBSV infected plant samples; lane D: Dual (CBSV and UCBSV) infected plant sample; Lanes E and F: UCBSV infected plant samples; lane G: Health control plant; lane NT: Non-template control; lane +ve: Dual (CBSV and UCBSV) infected positive plant sample; and L: 100 bp ladder (New England Biolabs).


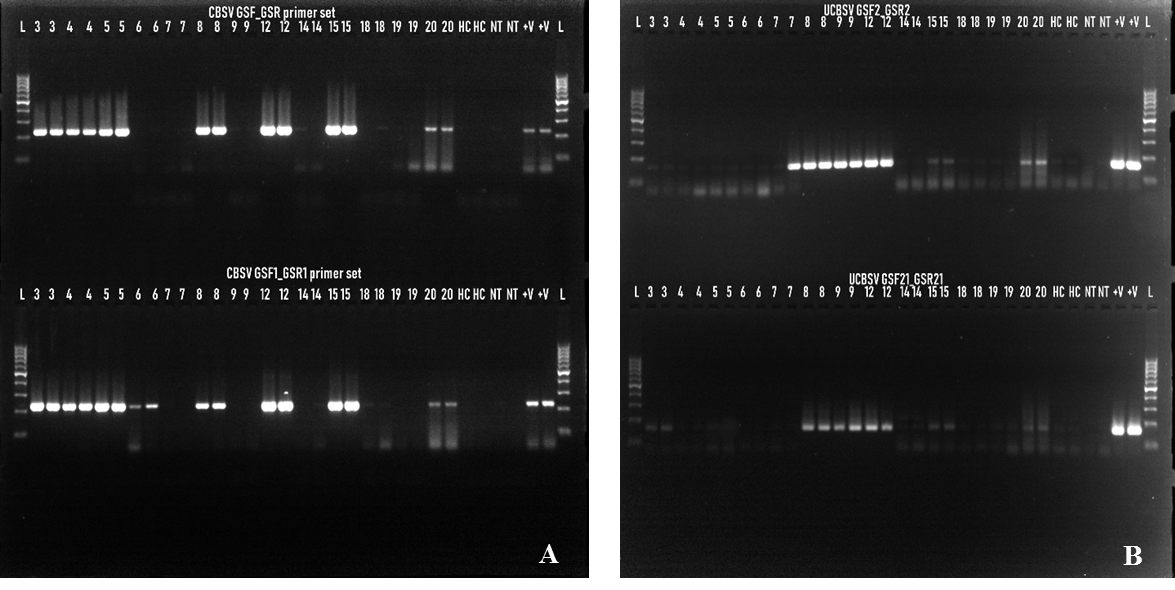


**Supplementary Figure S2.** A and B shows a gel indicating the screening of designed RPA primers targeting the cylindrcal inclusion (CI) region. Lanes 3 to 20, refer to the samples in Table 2 of the mauscript; lanes HC: Health control plant; lanes NT: Non-template control; lanes +ve: Dual (CBSV and UCBSV) infected positive plant sample; and lanes L: 100 bp ladder (New England Biolabs).


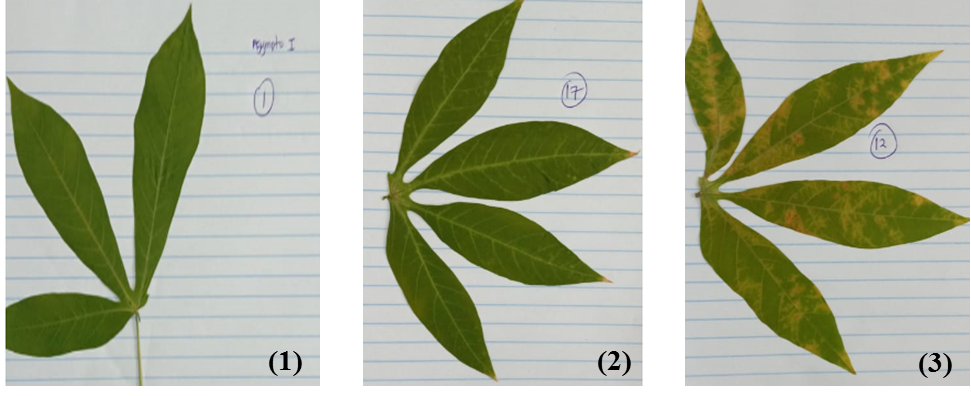


**Supplementary Figure S3:** Scale used n soring for cassava brown streak disease. (1) No visible symptoms of cassava brown streak disease (CBSD); (2) mild symptoms of CBSD; and (3) Severe symptoms of CBSD.

**References**

1. Adams IP, Abidrabo P, Miano DW, Alicai T, Kinyua Z, Clarke J, Macarthur R, Weeks R, Laurenson L, Hany U, Peters D, Potts M, Glover R, Boonham N, Smith J. 2013. High throughput real-time PCR assays for specific detection of Cassava brown streak disease causal viruses, and their application to testing of planting material. *Plant Pathology*, 62 (1):233-242.
2. Tomlinson JA, Ostoja-Starzewska S, Adams IP, Miano DW, Abidrabo, P, Kinyua Z, Alicai, T., Dickinson, M.J., Peters, D., Boonham, N. and Smith, J. 2013. Loop-mediated isothermal amplification for rapid detection of the causal agents of cassava brown streak disease. *Journal of Virological Methods*, 191:148–154.
3. Winter, S., Koerbler, M., Stein, B., Pietruszka, A., Paape, M., & Butgereitt, A. (2010). Analysis of cassava brown streak viruses reveals the presence of distinct virus species causing cassava brown streak disease in East Africa. *Journal of General Virology*, 91(5), 1365–1372.
